# Elevated SH3BP5 Correlates with Poor Outcome and Contributes to the Growth of Acute Myeloid Leukemia Cells

**DOI:** 10.3390/biom9090505

**Published:** 2019-09-19

**Authors:** Minjing Li, Shiyu Hao, Chunling Li, Huimin Xiao, Liyuan Sun, Zhenhai Yu, Naili Zhang, Yanlian Xiong, Dongmei Zhao, Yancun Yin

**Affiliations:** 1 Institute of Integrated Medicine, Binzhou Medical University, Yantai 264003, China; liminjing512@126.com; 2 School of Basic Medical Sciences, Binzhou Medical University, Yantai 264003, China; hsy1027125088@163.com (S.H.); bzyxylcl@163.com (C.L.); yzh78978@sohu.com (Z.Y.); znl-0@163.com (N.Z.); xyl8807@sina.com (Y.X.); 3 School of Pharmacy, Binzhou Medical University, Yantai 264003, China; xiaohuimin1129@163.com (H.X.); sunliyuan6062@163.com (L.S.)

**Keywords:** Acute myeloid leukemia, SH3BP5, Prognosis, Apoptosis, JNK

## Abstract

Current strategies are not especially successful in the treatment of acute myeloid leukemia (AML). The identification and characterization of oncogenes crucial to the survival and growth of leukemia cells will provide potential targets for the exploitation of novel therapies. Herein, we report that the elevated expression of SH3 domain-binding protein 5 (SH3BP5) significantly correlates with poor outcomes of AML patients. To test whether SH3BP5 contributes to the growth and survival of AML cells, we use the shRNA-encoding lentivirus system to achieve the knockdown of SH3BP5 expression in human AML cell lines U937, THP-1, Kasumi-1, and MV4-11. Functionally, the knockdown of SH3BP5 expression markedly inhibits the cell viability and induced apoptosis of these leukemia cells. Mechanistically, western blot analysis indicates that the knockdown of SH3BP5 expression decreases the phosphorylation of JNK and BAD. Moreover, the JNK agonist anisomycin rescues the growth inhibition phenotype of SH3BP5 deficiency in THP-1 cells. Moreover, the expression of SH3BP5 positively correlates with CD25 and CD123 levels. Finally, our study highlights the crucial role of SH3BP5 in promoting the survival of AML cells, and its suppression may be a potential therapeutic strategy for treating human AML.

## 1. Introduction

Despite the rapidly growing armamentarium of effective biologic agents and targeted therapies, AML remains a serious challenge for patients and hematologists. There were about 21,380 new diagnoses of AML in 2017 in USA, but conjected death cases were over 10,590, making AML the 6th highest cancer-related death in the male population [1]. Although a variety of treatment methods such as targeted therapy, immunotherapy, and stem cell transplantation have been established, they all have certain drawbacks and still cannot achieve ideal results. Therefore, it is imminent to continue to explore more effective methods for treating AML.

To identify novel therapeutic targets in human AML, we have established a systematic approach to explore potential genes important for leukemia propagation. Firstly, we determine new genes that correlate with the outcomes of AML patients using clinical databases. Then, we determine the biological function of the candidate genes using a loss-of-function approach in vitro. Furthermore, we validate the function of target candidate genes in leukemia mouse models in vivo. The candidate genes and their downstream signaling pathways will provide potential targets for the development of therapeutic approaches. We have identified several specific receptors, metabolizing enzymes, and some membrane proteins that support AML propagation using the above approach [2,3,4,5].

SH3BP5, a mitochondrial outer membrane scaffold protein, has a N-terminal SH3 domain binding site, a membrane spanning domain, and a two D-motif (KIM) on C-terminus [6,7]. The topology of SH3BP5 makes it crucial in JNK-mediated signal transduction to mitochondria and reactive oxygen species (ROS) production, and functional in mitochondrial dysfunction and cell death [8,9,10,11]. However, whether and how SH3BP5 regulates the propagation of AML remains unclear. Herein, we exhibit our findings that up-regulated expression of SH3BP5 is negatively related to the survival of AML patients. Notably, we find that SH3BP5 promotes the growth and survival of human AML cells, and c-Jun N-terminal kinases- Bcl-2 associated agonist of cell death (JNK-BAD) signaling is suppressed upon SH3BP5 knockdown.

## 2. Materials and Methods

### 2.1. Materials

Roswell Park Memorial Institute 1640 (RPMI-1640) and high glucose Dulbecco’s modified Eagle’s (DMEM) medium were purchased from Hyclone (South Logan, UT, USA). Fetal bovine serum (FBS) was purchased from Gibco (CA, USA). JNK agonist anisomycin was purchased from Tocris biosciences (Bristol, UK). Anti- SH3BP5 (#11127-2-AP), Caspase 3 (#19677-1-AP), JNK (#24164-1-AP), BAD (#10435-1-AP), and Actin (#60008-1-Ig) antibodies were purchased from Proteintech Group (Chicago, IL, USA). Anti- p-JNK Thr183/Tyr185 (#9255) and p-BAD Ser112 (#5284) antibodies were purchased from Cell Signaling Technology (Beverly, MA, USA).

### 2.2. Cell Culture

All the leukemia cell lines—MV4-11, THP-1, U937, Kasumi-1, Rch-Acv, Kasumi-2, and NALM-6—were cultured in PMI-1640 supplemented with 10% FBS and 1% pen/strep. HEK-293 T cells were maintained in high glucose DMEM supplemented with 10% FBS and 1% pen-strep. All cell lines were maintained in the humidified incubator containing 5% CO2 at 37 ℃. All the cell lines were kindly provided by Prof. Cheng Cheng Zhang (UT. Southwestern Medical Center).

### 2.3. Gene Expression Omnibus (GEO) and Clinical Databases

We analyzed gene expression datasets from human AML studies as previously described [4,12,13]. SH3BP5 mRNA levels in human AML and normal hematopoietic samples were determined from the GEO database (GSE6236, GSE1010, and GSE1159). The Cancer Genome Atlas (TCGA) dataset and RNAseq expression data from AML samples were acquired from the TCGA database (https://tcga-data.nci.nih.gov/tcga/). Transcriptomic profiles of SH3BP5 gene were measured in 200 primary AML patients. However, only 151 AML patients with intact clinical information were analyzed in this study.

### 2.4. Lentiviral shRNA Constructs, Production, and Infection

The targeting SH3BP5 shRNAs were designed online as we previously described [13]. The targeted oligonucleotide sequences of SH3BP5 were used as follows: shRNA 1: 5-GGCAAAGTACTATGTGCAG-3; shRNA2: 5-GCAAAAGGCGAGTACAAGA-3; shRNA3: 5-GCAACGGTGAAACTGGATGAA-3; shRNA4: 5-GATCTCAGATGAG ATCCACGA-3. The PLL3.7 lentiviral vector was used to express designed SH3BP5 shRNAs. Lentiviral production and cells infection were performed essentially as previous described [4,14]. For lentiviral production, HEK-293T cells were grown on 10-cm culture plates to ~90% confluence. Scramble or SH3BP5 shRNA constructs were mixed with psPAX2 and pMD2G (7.5 µg:5.5 µg:2 µg), respectively. The mixed solution was incubated for 15 min, then transfected into HEK-293T cells using polyJet. At 6 h post-transfection, the supernatant was discarded and replaced with fresh DMEM. 48 h later, the lentivirus-containing supernatant was collected and filtered with a pore filter. For infection, U937 or THP-1 cells were seeded in 6-well plate and grown to ~50% confluence. Cells were maintained in lentivirus supernatant and centrifuged at 2000 rpm for 120 min at 37 °C. After continued cultured in lentiviral supernatant for 4 h, the lentivirus supernatant was discarded and replaced with RPMI-1640 medium with 10% FBS. 24 h later, the infection procedures were repeated for the secondary infection.

### 2.5. Quantitative Real-Time RT-PCR

Real-time (RT) PCR primers were designed online as previously described [13]. Briefly, total RNA was extracted from leukemia cells using TRIzol, and cDNA was synthesized. RT-PCR analyses were carried out using SYBR-Green PCR Master Mix (Toyobo, Osaka, Japan). The relative expression level of SH3BP5 mRNA was calculated using ΔΔCt methods after being normalized to GAPDH. The experiments were repeated three times.

### 2.6. Cell Growth and Apoptosis Assays

For cell growth assay, lentivirus-infected cells (GFP positive) were sorted by flow cytometry at 48 h post-infection. Then, cells were seeded in 24-well plates at 10,000 cells per well in triplicate wells. Then, cell numbers were counted at 2, 4, and 6 days. Cell apoptosis was evaluated using the Annexin V-PE/7-AAD apoptosis detection kit (#559783, BD Bioscience, San Jose, CA), as previously described [4]. All the experiments were performed three times.

### 2.7. Western Blot

Cells were harvested and lysed in cold RIPA lysis buffer containing protease and phosphatase inhibitor cocktails. The proteins were resolved by SDS-PAGE and then transferred to a PVDF membrane via the permeant blotting method. Then, the membrane was blocked with 5% BAS and incubated with appropriate primary antibodies at 4 °C overnight. Thereafter, the membranes were incubated with the appropriate HRP-conjugated 2ed antibodies after being washed three times. Detection was performed with ECL chemiluminescent agent. Densitometric analysis of blots were performed using Image J. The levels of protein expression were normalized to Actin.

### 2.8. Statistical Analyses

All statistical analyses were performed with the SPSS 24.0 software package. Difference between SH3BP5 expression and cell growth/apoptosis was compared using two-tailed student’s t-test or two-way Anova. Associations between SH3BP5 expression and clinicopathologic parameters were analyzed with a chi-square test. Overall, survival curves were determined by the Kaplan–Meier technique and contrasted via log-rank test. Univariate and multivariate Cox analyses were used to determine risk factors for overall survival. For all experiments, differences were considered statistically significant if p < 0.05.

## 3. Results

### 3.1. Expression of SH3BP5 in AML and Its Connection to AML Pathological Characteristics

**Figure 1:**
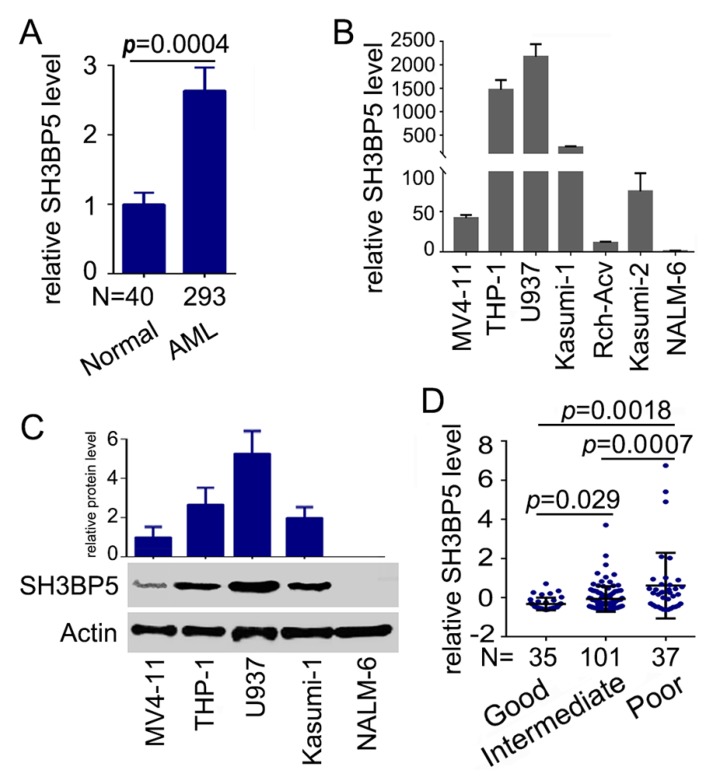
Elevated expression of SH3BP5 in acute myeloid leukemia (AML) cells and patients. (**A**) Relative expression of SH3BP5 mRNA in human normal and acute myeloid leukemia samples (GSE6236, GSE1010, and GSE1159). (**B**) SH3BP5 mRNA expression as determined by RT-PCR in human AML and B-ALL cell lines. Fold changes of SH3BP5 to that in ANLM-6 cells were plotted. N = 3. (**C**) Expression of SH3BP5 in AML cell lines was analyzed by western blot. Densitometric analysis of SH3BP5 normalized to actin was presented as a fold change compared with the value in MV4-11 cells. N = 3. (**D**) mRNA levels of SH3BP5 in human AML patients with good, intermediate, and poor karyotype.

To investigate the expression profile of SH3BP5 in human AML cells, microarray data derived from the GEO database (GSE6236, GSE1010, and GSE1159) were analyzed. The results suggested that expression of SH3BP5 in AML patients was significantly higher than that in healthy samples (Figure 1A). Further, the mRNA levels of SH3BP5 in leukemia cell lines were analyzed by quantitative RT-PCR. Results showed that SH3BP5 mRNA is highly expressed in AML cell lines THP-1, U937, Kasumi-1, and MV4-11 (Figure 1B). SH3BP5 was also highly expressed in the lymphoblastic leukemia (ALL) cell line Kasumi-2; however, it was expressed in Rch-Acv and NALM-6 at lower levels (Figure 1B). Concordantly, western blot results demonstrated that the protein level of SH3BP5 was significantly higher in AML cell lines than that in ALL NALM-6 cell line (Figure 1C). Moreover, the expression of SH3BP5 was further studied in an independent cohort of AML patients comprising of good, intermediate, and poor karyotypes. Interestingly, expression of SH3BP5 was significantly up-regulated in AML patients with poor karyotype compared to that in patients with good or intermediate karyotypes (Figure 1D). Together, these results suggested that SH3BP5 was highly expressed in AML cells. In-depth patient attributes and the correlations between SH3BP5 expression and clinicopathological features are included in Table 1. The data revealed that high expression of SH3BP5 was significantly related to age (p = 0.0152), cytogenetics risk (p = 0.0278), and karyotypes (p = 0.0117) of AML patients, and it was a trend toward FAB classifications (p = 0.0801). However, SH3BP5 expression was not related to other clinical characteristics (p > 0.1).

### 3.2. High Expression of SH3BP5 is Connected to Worse Survival and is a Separate Indicator of Poor Survival in AML Patients

**Figure 2:**
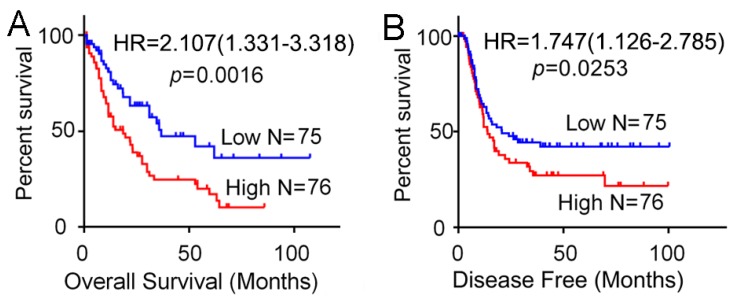
Elevated expression of SH3BP5 inversely correlated with survival of AML patients. (**A**) Overall and (**B**) disease-free survival of AML patients relative to SH3BP5 mRNA was determined by Kaplan–Meier analysis. Patients were divided into two groups based on expression levels of SH3BP5 above or below the 50th percentile. (A-B, data from the TCGA AML database).

**Figure 3:**
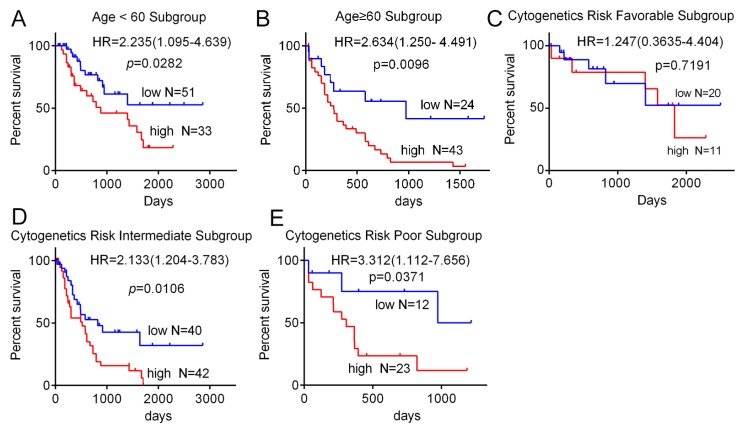
Relationship between SH3BP5 expression and age or cytogenetics risk in AML patients. Kaplan–Meier analysis of overall survival in patients with age < 60 (**A**) or age ≥ 60 (**B**) subgroup patients according to SH3BP5 expression level. Kaplan–Meier analysis of overall survival in patients with cytogenetics risk-favorable (**C**), intermediate (**D**), and poor (**E**) subgroup patients according to SH3BP5 expression level. p value was calculated by log-rank test.

To further investigate the potential function of SH3BP5 on AML propagation, Kaplan–Meier analysis of the relationship between SH3BP5 expression level and clinical endpoints of AML patients was performed. Analysis demonstrated that the higher expression of SH3BP5 was significantly associated with shorter overall/disease free survival in AML patients (Figure 2A,B). To examine if this discovery was separate from well-established prognostic indicators, such as age, cytogenetics risk, and karyotypes, we conducted Cox proportional hazard analyses of each of the clinicopathological variables with SH3BP5 expression. Univariate Cox analysis showed that age (HR = 2.615, 95% CI: 1.906–4.927, p = 0.0001), FAB classifications (p = 0.0001), Karyotypes (p = 0.0165), and SH3BP5 expression (HR = 2.107, 95% CI:1.331–3.318, p = 0.0035) were associated with overall survival of AML patients (Table 2). After that, multivariate Cox regression analysis, including potential risk factors determined by univariate Cox analysis, was conducted. In addition to age and cytogenetics risk, SH3BP5 expression was shown to be an independent prognostic factor for overall survival of AML patients (HR = 2.020, 95% CI:1.271–3.215, p = 0.0029) (Table 2). Consistently, the overall survival rates were significantly different between SH3BP5-high and SH3BP5-low patients both in age < 60 (HR = 2.235, p = 0.0282) and age ≥ 60 (HR = 2.634, p = 0.0096) subgroups (Figure 3A,B). Moreover, in cytogenetics risk-intermediate (HR = 2.133, p = 0.0106) and cytogenetics risk-poor (HR = 3.312, p = 0.0371) subgroups, the expression of SH3BP5 also inversely correlated with the overall survival of AML patients (Figure 3C–E). Together, these results suggested that expression of SH3BP5 may be an independent prognostic indicator in AML patients.

### 3.3. Knockdown of SH3BP Inhibits Cell Viability and Induces Apoptosis of AML Cells

**Figure 4:**
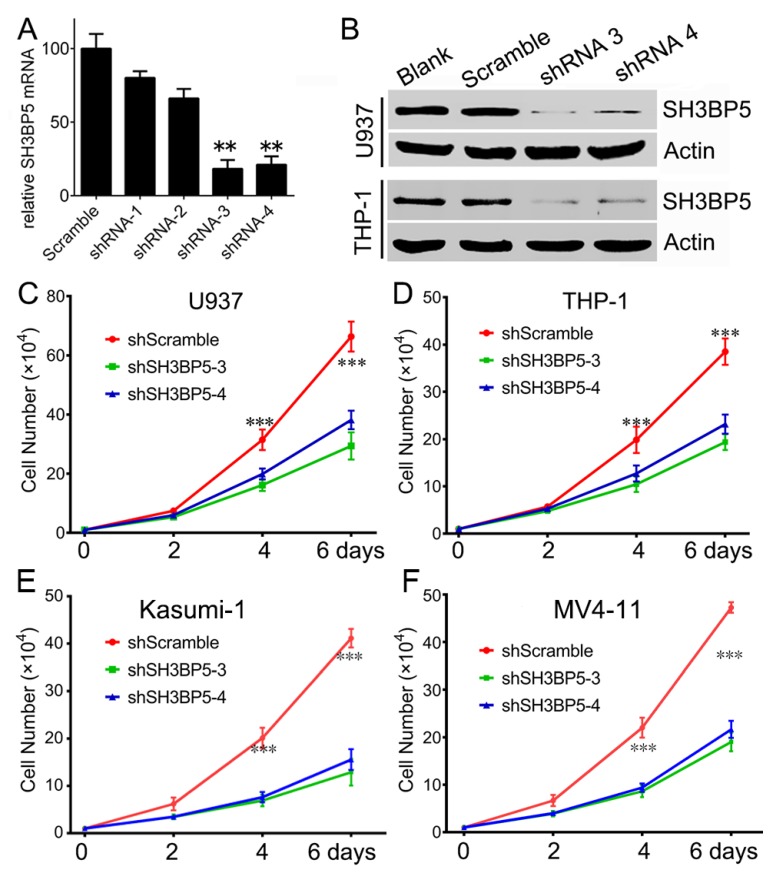
Knockdown of SH3BP5 expression suppresses growth of U937 and THP-1 cells. (**A**) U937 cells were infected with SH3BP5 or scramble shRNA for 24 h followed by RT-PCR analysis transcription of SH3BP5. N = 5, **, p < 0.01 compared to scramble shRNA group. (**B**) U937 or THP-1 cells were infected with indicated shRNAs for 72 h followed by western blot analysis expression of SH3BP5. (**C**–**F**) Cell numbers of U937, THP-1, Kasumi-1, and MV4-11, at 2, 4, and 6 days post-infection, with indicated shRNA. N = 3, ***, p < 0.005 compared with the SH3BP5 shRNA groups.

**Figure 5:**
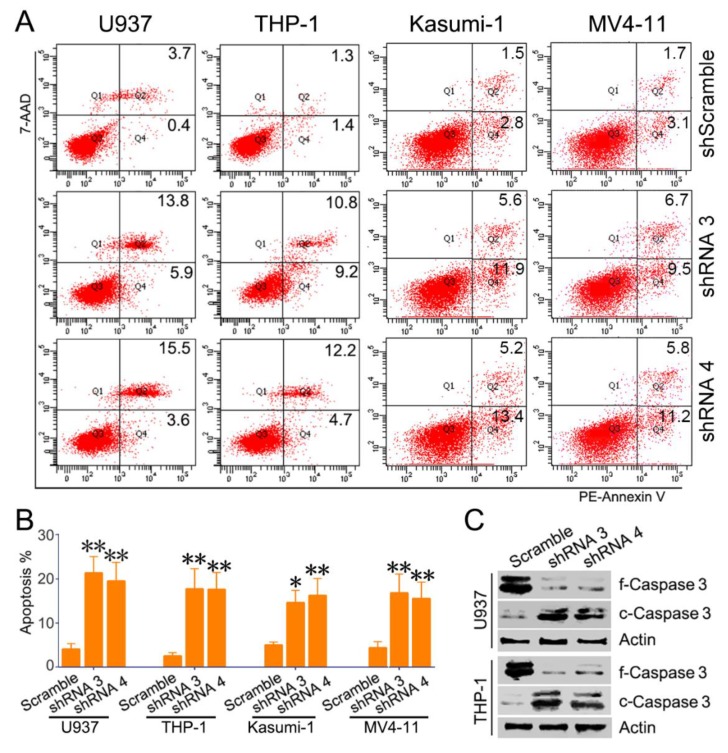
Knockdown of SH3BP5 expression-induced apoptosis of AML cells. (**A**) U937, THP-1, Kasumi-1, and MV4-11 cells were infected with SH3BP5 or scramble shRNAs for 72 h, followed by analysis of cell apoptosis by 7-AAD and PE-Annexin V labeling. Shown are representative scatter plots of flow cytometry. (**B**) Shown is apoptosis rate of U937, THP-1, Kasumi-1, and MV4-11 cells. N = 3, *, p < 0.05 **, and p < 0.01 compared with the scramble shRNA group. (**C**) U937 and THP-1 cells were infected with SH3BP5 or scramble shRNAs for 72 h, followed by western blotting analysis of expression of Caspase 3 (f-Caspase 3, full-length Caspase 3; c-Caspase 3, cleaved-Caspase 3).

To explore the potential role of SH3BP5 in AML, expression of SH3BP5 was knocked down in AML cells using lentivirus-encoded shRNAs. Firstly, the efficiency of the designed shRNAs targeted SH3BP5 was examined. RT-PCR results suggested that shRNA 3 and 4 efficiently inhibit SH3BP5 expression at mRNA level compared to scramble shRNA (Figure 4A). We further confirmed the efficiency of shRNA3 and shRNA4 by western blot. Consistently, shRNA3 and shRNA4 could efficiently inhibit SH3BP5 expression at protein level both in U937 and THP-1 cells (Figure 4B). In subsequent experiments, shRNA3 or shRNA4 was transfected to inhibit expression of SH3BP5. To further evaluated the biology function of SH3BP5, the cell count assay was performed. Results showed that knockdown of SH3BP5 expression significantly decreased growth of U937 cells. Growth of U937 cells transfected with SH3BP5 shRNAs was much slower at 2 days and was more apparent at 4 days post-transfection compared to cells transfected with scramble shRNAs (Figure 4C). Knockdown of SH3BP5 expression also resulted in a sharply decline in cell growth of THP-1, Kasumi-1, and MV4-11 cells (Figure 4D–F). To investigate the underlying mechanisms of SH3BP5 supporting growth of AML cells, cell proliferation and apoptosis status was analyzed. Knockdown of SH3BP5 expression has no effect on cell cycle distribution; however, SH3BP5 knockdown dramatically increased cell apoptosis rate of U937, THP-1, Kasumi-1, and MV4-11 cells (Figure 5A,B). For instance, the shRNA3- or shRNA4-infected U937 cells had apoptosis rates of 21.3 ± 3.7% and 19.5 ± 4.2% respectively, compared to 4.1 ± 1.2% of scrambled shRNA-treated cells (Figure 5B). Moreover, knockdown of SH3BP5 expression significantly decreased full-length Caspase 3 and increased cleaved Caspase 3 expression in U937 and THP-1 cells (Figure 5C). This result further supports the conclusion that inhibiting SH3BP5 expression induces apoptosis of AML cells. Together, these results suggest that SH3BP5 contributes to leukemia cell growth and survival.

### 3.4. SH3BP5/JNK/BAD Signaling Regulates Survival of AML Cells

**Figure 6:**
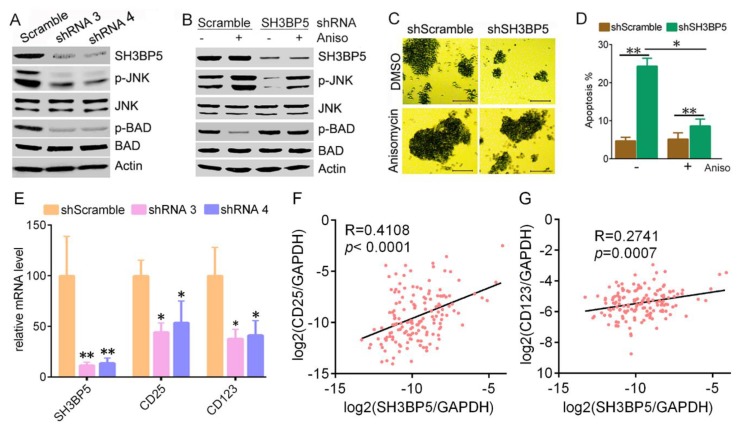
SH3BP5 knockdown inhibited activation of JNK-BAD signaling and expression of CD25, CD123. (**A**) THP-1 cells were infected with SH3BP5 or scramble shRNAs for 48 h, followed by western blotting analysis of phosphorylation of JNK and BAD. (**B**–**D**), THP-1 cells were pretreated with JNK agonist anisomycin (5 μM) for 4 h, then were infected with SH3BP5 or scramble shRNAs. (**B**), western blotting analysis phosphorylation of JNK and BAD at 2 days post-transfection. (**C**), shown are representative images of THP-1 cells at 4 days post-transfection. Bars, 50 μm. (**D**), flow cytometry analysis apoptosis at 3 days post-transfection. Shown is apoptosis rate. *, p < 0.01; **, p < 0.001. (**E**), THP-1 cells were infected with SH3BP5 or scramble shRNA for 24 h followed by RT-PCR analysis transcription of indicated genes. N = 4, *, p < 0.01; **, p < 0.01 compared to scramble shRNA group. **F**–**G**, Correlations between SH3BP5 and CD25, CD123 expression in 151 AML patients. The log2 transcription level of SH3BP5, CD25, and CD123 was shown after normalized to GAPDH.

Previous studies have suggested that SH3BP5 physically interacts with JNK by its N-terminal SH3 domain binding site, and plays unique roles in JNK-mediated signal transduction [6,7,8,9]. We hypothesized that JNK-mediated signaling may be involved in function of SH3BP5 to promote AML cells survival. Herein, we found that knockdown of SH3BP5 expression by shRNAs decreased phosphorylation of JNK in THP-1 cells. Levels of p-BAD, which is a target of JNK, were also significantly decreased in SH3BP5 knockdown cells (Figure 6A). Consistently, levels of p-JNK and p-BAD were increased in THP-1 cells that were pre-treated with JNK agonist anisomycin (Figure 6B). These data imply that SH3BP5 promotes the activation of JNK-BAD signaling. To test whether JNK-BAD signaling is involved in the SH3BP3-promoted survival of AML cells, THP-1 cells were pre-treated with JNK agonist anisomycin, then infected with SH3BP5 shRNA. Results showed that anisomycin slightly induced cell growth in scrambled shRNA-treated cells. SH3BP5 knockdown significantly decreased growth of THP-1 cells. Interestingly, anisomycin rescued the inhibition of growth by SH3BP5 knockdown (Figure 6C). Moreover, anisomycin caused THP-1 cell apoptosis similar to levels of untreated cells, further indicating that activated JNK rescued the SH3BP5 deficiency (Figure 6D). Together, these data indicated that the oncogenic function of SH3BP5 may be mediated by the JNK-BAD signaling pathway. Leukemia stem cells play prominent roles in leukemogenesis and propagation due to their capacities of self-renewal, proliferation, and differentiation. Thus, the established markers of leukemia stem cells such as CD25 and CD123 were determined. The RT-PCR result showed that knockdown of SH3BP5 expression decreased transcription of CD25 and CD123 (Figure 6E). Conformably, Spearman’s rank tests showed that the mRNA expression of SH3BP5 was positively correlated with the mRNA expression of CD25 and CD123 in 151 AML patients (Figure 6F,G).

## 4. Discussion

Herein, our data revealed that elevated expression of SH3BP5 significantly correlated with poor survival of AML patients. Moreover, SH3BP5 was highly expressed in many AML cell lines both at mRNA and protein levels. Using a loss-of-function approach, we further reported that SH3BP5 promoted AML propagation through suppressing cells apoptosis.

SH3BP5, as a novel JNK-interacting protein, was associated with disturbed mitochondrial function and elevated mitochondrial ROS production [8]. Recent studies have shown that SH3BP5 plays significant roles in sustained JNK activation. Briefly, activated JNK triggered by physical and chemical stress translocates to mitochondria and interacts with SH3BP5, leading to inhibition of mitochondrial electron transport chain and thus production of ROS, which further activates JNK. Thus, the JNK-SH3BP5-ROS loop drives sustained activation of JNK. However, function of SH3BP5 in AML development is still largely unknown, and more and more studies have found that the JNK signaling is closely related to AML. For instance, recombinant human thrombomodulin promoted differentiation and growth arrest of THP-1 cells via activating JNK/c-Jun signaling pathway [15]. Moreover, TNF stimulation stimulates the JNK pathway, leading to the up-regulated expression of anti-apoptotic genes in leukemia cells [16]. Our present study suggested that SH3BP5 may support AML cells growth via activating JNK signaling. This is the first demonstration of the SH3BP5 function in leukemia propagation and in the hematogenic system. Our study might lead to a progression in the field of SH3BP5 function in promoting leukemia cell growth by JNK signaling pathway. Notably, novel inhibitors targeting SH3BP5 may hold promise for human leukemia treatment, due to the lack of obvious physical defect of the SH3BP5 knockout mice [17]. It may be beneficial to study the potential roles of SH3BP5 in different types of leukemia.

JNK-mediated pathways regulate a wide range of developmental function depending on the cell type and lineage, although they are somewhat contradictory [18,19]. On the one hand, JNK contributes to cancer cell apoptosis. JNK signaling plays crucial roles in taurine-induced apoptosis of colorectal cancer cells [20]. Further, endoplasmic reticulum stress induced interaction of JNK with mitochondrial SH3BP5, leading to impaired respiration and increased mitochondrial ROS, sustaining JNK activation culminating in apoptosis [8]. Contradictory, our results suggested that SH3BP5-mediated activation of JNK contributes to survival of cancer cells. We hypothesized that knockdown of SH3BP5 expression decreased phosphorylation of JNK, thus decreasing phosphorylation of the pro-apoptotic protein BAD. Dephosphorylated BAD interacted with the anti-apoptotic molecule BCL-XL, thus promoting apoptosis of AML cells. Consistent with our study, Lin, X. et al found that activated JNK/c-JUN signaling induced by elevated HO-1 suppressed the apoptosis of AML cells [21]. Moreover, JNK is required for IL-3-induced cell survival by phosphorylation and inactivation of the pro-apoptotic Bcl-2 family protein BAD [22].

The opposing functions of JNKs attributed to the findings that JNKs activate different substrates, based on cell type temporal aspects, or specific stimulus [23]. Evidence suggested that whether a cell undergoes survival or apoptosis in response to the JNK signaling pathway is determined by the duration of JNK activation. Sustained JNK activation (> 2 h) induces cell death, whereas transient JNK activation (< 2 h) promotes cell survival [24]. In addition, Papa et al. showed that the pro-survival activity of JNK was dependent on NF-κB signaling pathway. Suppression of the NF-κB signaling converted the pro-survival activity of JNK to pro-apoptosis activity [25]. In the present study, we found that SH3BP5-mediated activation JNK contributes to survival of cancer cells. In spite of the opposing roles of JNK, further studies are urgently needed to ascertain the different functions of JNKs in specific conditions, such as stimulus, cell context, specific substrates, and different cancer types.

## 5. Conclusions

In conclusion, we found that expression of SH3BP5 was enhanced in AML cells and was negatively correlated with survival in AML patients. Elevated expression of SH3BP5 was an independent prognostic factor for AML patients. Furthermore, SH3BP5-mediated activation of JNK-BAD signaling contributes to the survival of AML cells. Taken together, our data suggested that SH3BP5 played vital roles in progression of AML and demonstrated SH3BP5 might be a potential prognostic factor and treatment target for human AML.

## Figures and Tables

**Table 1:** Association between SH3BP5 expression and clinicopathological characteristics in AML patients.

Patient’s Parameters	SH3BP5 Low, 75	SH3BP5 High, 76	p
Sex, male/female	42/33	41/35	0.7999
Median age, years (range)	51 (21–81)	61(25–88)	0.0152 *
FAB classifications ^1^			0.0801
M0	5	10	
M1	20	15	
M2	25	13	
M3	6	9	
M4	14	15	
M5	4	11	
M6	0	2	
M7	0	1	
Not Classified	1	0	
Cytogenetics Risk			0.0278 *
Favorable	20	11	
Intermediate	40	42	
Poor	12	23	
No data	3	0	
Karyotypes			0.0117 *
Normal	36	36	
t (8;21)	7	0	
t (15;17)	5	7	
complex	4	15	
others	15	10	
No data	8	8	
Gene mutations			
FLT3 mutation P/N	19/56	28/48	0.1267
RAS expression P/N	5/70	4/72	0.7157
NPM1 expression P/N	13/62	21/55	0.1298
IDH1 expression P/N	10/65	16/60	0.2090

^1^ French–American–British (FAB) classification; * represents p values with significant difference.

**Table 2:** Univariate and multivariate Cox regression analysis of overall survival in AML patients; * represents p values with significant difference.

Characteristics	HR	Univariate 95% CI	p	HR	Multivariate 95% CI	p
Sex (female vs. male)	1.006	0.644–1.573	0.9781			
Age (≥60 vs. <60)	2.615	1.906–4.927	0.0001 *	2.645	1.655–4.228	0.0001 *
FAB classifications	///	///	0.0001 *	///	///	0.1316
Cytogenetics risk	///	///	0.1599	1.487	1.043–2.121	0.0285 *
Karyotypes	///	///	0.0165 *	///	///	0.7959
FLT3 (mutation vs. normal)	1.328	0.8237–2.234	0.2344			
NPM1 (positive vs. negative)	0.5966	0.3821–1.021	0.0647			
IDH1 (positive vs. negative)	0.7025	0.4116–1.278	0.2707			
SH3BP5 (high vs. low)	2.107	1.331–3.318	0.0035 *	2.020	1.271–3.215	0.0029 *

## References

[1] Siegel R.L., Miller K.D., Jemal A. (2017). Cancer Statistics, 2017. CA Cancer J. Clin..

[2] Li C., Xie J., Lu Z., Xie J., Lu Z., Li M., Wang Y., Zhang C.C. (2015). ADCY7 supports development of acute myeloid leukemia. Biochem. Biophys. Res. Commun..

[3] Chen C., Yin Y., Li C., Chen J., Xie J., Lu Z., Li M., Wang Y., Zhang C.C. (2016). ACER3 supports development of acute myeloid leukemia. Biochem. Biophys. Res. Commun..

[4] Chen J., Li C., Zhan R., Yin Y. (2018). SPG6 supports development of acute myeloid leukemia by regulating BMPR2-Smad-Bcl-2/Bcl-xl signaling. Biochem. Biophys. Res. Commun..

[5] Kang X., Lu Z., Cui C., Deng M., Fan Y., Dong B., Han X., Xie F., Tyner J.W., Coligan J.E. (2015). The ITIM-containing receptor LAIR1 is essential for acute myeloid leukaemia development. Nat. Cell Biol..

[6] Wiltshire C., Matsushita M., Tsukada S., Gillespie D.A., May G.H. (2002). A new c-Jun N-terminal kinase (JNK)-interacting protein, Sab (SH3BP5), associates with mitochondria. Biochem. J..

[7] Wiltshire C., Gillespie D.A., May G.H. (2004). Sab (SH3BP5), a novel mitochondria-localized JNK-interacting protein. Biochem. Soc. Trans..

[8] Win S., Than T.A., Fernandez-Checa J.C., Kaplowitz N. (2014). JNK interaction with Sab mediates ER stress induced inhibition of mitochondrial respiration and cell death. Cell Death Dis..

[9] Win S., Than T.A., Le B.H., Garcia-Ruiz C., Fernandez-Checa J.C., Kaplowitz N. (2015). Sab (Sh3bp5) dependence of JNK mediated inhibition of mitochondrial respiration in palmitic acid induced hepatocyte lipotoxicity. J. Hepatol..

[10] Win S., Than T.A., Kaplowitz N. (2018). The Regulation of JNK Signaling Pathways in Cell Death through the Interplay with Mitochondrial SAB and Upstream Post-Translational Effects. Int. J. Mol. Sci..

[11] Win S., Than T.A., Zhang J., Oo C., Min R.W.M., Kaplowitz N. (2018). New insights into the role and mechanism of c-Jun-N-terminal kinase signaling in the pathobiology of liver diseases. Hepatology.

[12] Li M., Jin C., Xu M., Zhou L., Li D., Yin Y. (2017). Bifunctional enzyme ATIC promotes propagation of hepatocellular carcinoma by regulating AMPK-mTOR-S6 K1 signaling. Cell Commun. Signal..

[13] Li M., Gao J., Li D., Yin Y. (2018). CEP55 Promotes Cell Motility via JAK2(-)STAT3(-)MMPs Cascade in Hepatocellular Carcinoma. Cells.

[14] Yin Y., Zhou L., Zhan R., Zhang Q., Li M. (2018). Identification of WDR12 as a novel oncogene involved in hepatocellular carcinoma propagation. Cancer Manag. Res..

[15] Yang J., Ikezoe T., Nishioka C., Honda G., Yokoyama A. (2012). Thrombomodulin-induced differentiation of acute myelomonocytic leukemia cells via JNK signaling. Leuk. Res..

[16] Volk A., Li J., Xin J., You D., Zhang J., Liu X., Xiao Y., Breslin P., Li Z., Wei W. (2014). Co-inhibition of NF-kappaB and JNK is synergistic in TNF-expressing human AML. J. Exp. Med..

[17] Win S., Than T.A., Min R.W., Aghajan M., Kaplowitz N. (2016). JNK mediates mouse liver injury through a novel Sab (SH3BP5)-dependent pathway leading to inactivation of intramitochondrial Src. Hepatology.

[18] Bode A.M., Dong Z. (2007). The functional contrariety of JNK. Mol. Carcinog..

[19] Chen F. (2012). JNK-induced apoptosis, compensatory growth, and cancer stem cells. Cancer Res..

[20] Liu Z., Xia Y., Zhang X., Liu L., Tu S., Zhu W., Yu L., Wan H., Yu B., Wan F. (2018). Roles of the MST1-JNK signaling pathway in apoptosis of colorectal cancer cells induced by Taurine. Libyan J. Med..

[21] Lin X., Fang Q., Chen S., Liu L., Tu S., Zhu W., Yu L., Wan H., Yu B., Wan F. (2015). Heme oxygenase-1 suppresses the apoptosis of acute myeloid leukemia cells via the JNK/c-JUN signaling pathway. Leuk. Res..

[22] Yu C., Minemoto Y., Zhang J., Liu J., Tang F., Bui T.N., Xiang J., Lin A. (2004). JNK suppresses apoptosis via phosphorylation of the proapoptotic Bcl-2 family protein BAD. Mol. Cell.

[23] Dhanasekaran D.N., Reddy E.P. (2017). JNK-signaling: A multiplexing hub in programmed cell death. Genes Cancer.

[24] Ventura J.J., Hubner A., Zhang C., Liu J., Tang F., Bui T.N., Xiang J., Lin A. (2006). Chemical genetic analysis of the time course of signal transduction by JNK. Mol. Cell.

[25] Papa S., Zazzeroni F., Bubici C., Jayawardena S., Alvarez K., Matsuda S., Nguyen D.U., Pham C.G., Nelsbach A.H., Melis T. (2004). Gadd45 beta mediates the NF-kappa B suppression of JNK signalling by targeting MKK7/JNKK2. Nat. Cell Biol..

